# Design, synthesis and biological evaluation of 3-(2-aminooxazol-5-yl)-2*H*-chromen-2-one derivatives

**DOI:** 10.1186/s13065-018-0499-x

**Published:** 2018-12-04

**Authors:** Saloni Kakkar, Sanjiv Kumar, Siong Meng Lim, Kalavathy Ramasamy, Vasudevan Mani, Syed Adnan Ali Shah, Balasubramanian Narasimhan

**Affiliations:** 1 Faculty of Pharmaceutical Sciences, Maharshi Dayanand University, Rohtak, 124001 India; 2 Faculty of Pharmacy, Universiti Teknologi MARA (UiTM), Puncak Alam Campus, 42300 Bandar Puncak Alam, Selangor Darul Ehsan Malaysia; 3 Collaborative Drug Discovery Research (CDDR) Group, Pharmaceutical Life Sciences Community of Research, Universiti Teknologi MARA (UiTM), 40450 Shah Alam, Selangor Darul Ehsan Malaysia; 4 Department of Pharmacology and Toxicology, College of Pharmacy, Qassim University, Buraidah, 51452 Kingdom of Saudi Arabia; 5 Atta-ur-Rahman Institute for Natural Products Discovery (AuRIns), Universiti Teknologi MARA (UiTM), PuncakAlam Campus, 42300 Bandar Puncak Alam, Selangor Darul Ehsan Malaysia

**Keywords:** Oxazole, Synthesis, Antimicrobial, Anticancer, Characterization

## Abstract

**Background:**

In view of wide range of biological activities of oxazole, a new series of oxazole analogues was synthesized and its chemical structures were confirmed by spectral data (Proton/Carbon-NMR, IR, MS etc.). The synthesized oxazole derivatives were screened for their antimicrobial and antiproliferative activities.

**Results and discussion:**

The antimicrobial activity was performed against selected fungal and bacterial strains using tube dilution method. The antiproliferative potential was evaluated against human colorectal carcinoma (HCT116) and oestrogen- positive human breast carcinoma (MCF7) cancer cell lines using Sulforhodamine B assay and, results were compared to standard drugs, 5-fluorouracil and tamoxifen, respectively.

**Conclusion:**

The performed antimicrobial activity indicated that compounds **3**, **5**, **6**, **8** and **14** showed promising activity against selected microbial species. Antiproliferative screening found compound **14** to be the most potent compound against HCT116 (IC_50_ = 71.8 µM), whereas Compound **6** was the most potent against MCF7 (IC_50_ = 74.1 µM). Further, the molecular docking study has been carried to find out the interaction between active oxazole compounds with CDK8 (HCT116) and ER-α (MCF7) proteins indicated that compound **14** and **6** showed good dock score with better potency within the ATP binding pocket and may be used as a lead for rational drug designing of the anticancer molecule. 
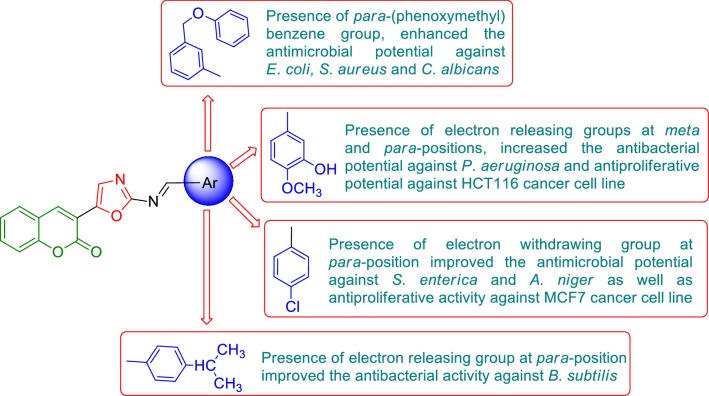

## Background

**Fig. 1:**
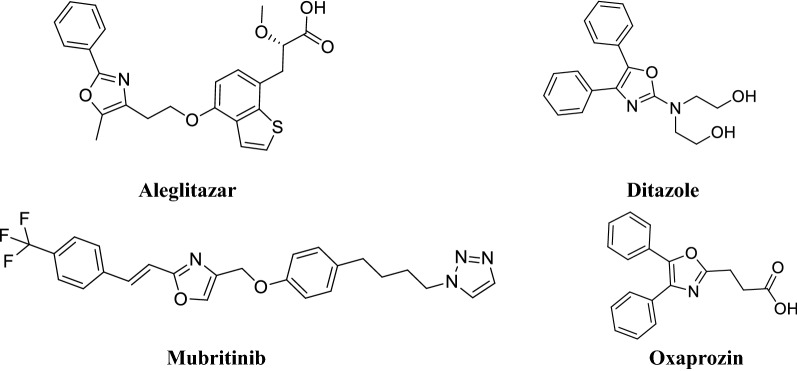
Marketed drugs containing oxazole

Multidrug resistance and emergence of new infectious diseases are amongst the major challenges in the treating of microbial infections which necessitates the discovery of newer antimicrobial agents [1]. Cancer is one of the serious health issues and many more novel anticancer agents are needed for effective treatment of cancer [2, 3]. Heterocyclic compounds offer a high degree of structural diversity and have proven to be broadly and economically useful as therapeutic agents like benzoxazole [4, 5], indole [3], Quinoline-Branched Amines [6, 7], pyrimidine analogues [8]. The oxazole moiety is reported to have broad range of biological potential such as anti-inflammatory, analgesic, antibacterial [9], antifungal [10], hypoglycemic [11], antiproliferative [12], antitubercular [13], antiobesity [14], antioxidant [15], antiprogesteronic [16], prostacyclin receptor antagonist [17], T-type calcium channel blocker [18] and transthyretin (TTR) amyloid fibril inhibitory activities [19]. A number of marketed drugs (Fig. 1) are available in which oxazole is the core active moiety such as aleglitazar (antidiabetic) [20], ditazole (platelets aggregation inhibitor) [21], mubritinib (tyrosine kinase inhibitor) [22], and oxaprozin (COX-2 inhibitor) [23].

Molecular docking studies provide the most detailed possible view of drug-receptor interaction and have created a new rational approach to drug design. The CDKs (cyclin dependent kinase) is an enzyme family that plays an important role in the regulation of the cell cycle and thus is an especially advantageous target for the development of small inhibitory molecules. Selective inhibitors of the CDKs can be used for treating cancer or other diseases that cause disruptions of cell proliferation [24]. Estrogen receptor alpha (ERα) is the major driver of ~ 75% of all breast cancers. Current therapies for patients with ER+ breast cancer are largely aimed at blocking the ERα signaling pathway. For example, tamoxifen blocks ERα function by competitively inhibiting E2/ERα interactions and fulvestrant promotes ubiquitin-mediated degradation of ERα. Endocrine therapies are estimated to have reduced breast cancer mortality by 25 ± 30% [25].

**Fig. 2:**
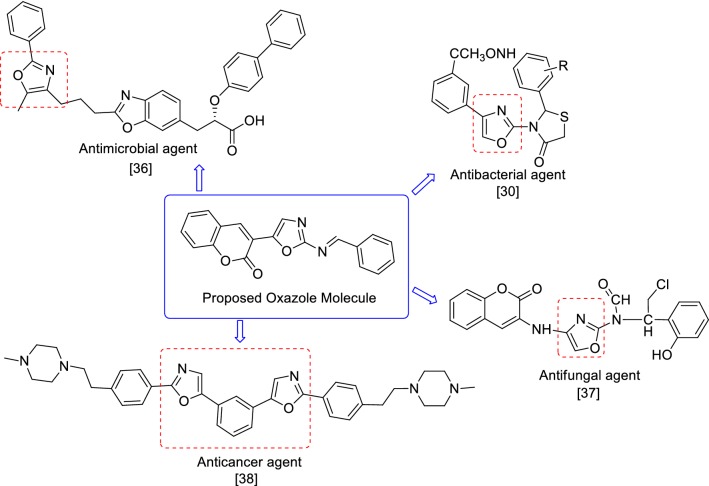
Biological profile of oxazole derivatives

On the basis of the information obtained from literature survey (Fig. 2), in the present work we hereby report the synthesis, antimicrobial and antiproliferative potentials of oxazole derivatives.

## Results and discussion

### Chemistry

**Scheme 1:**
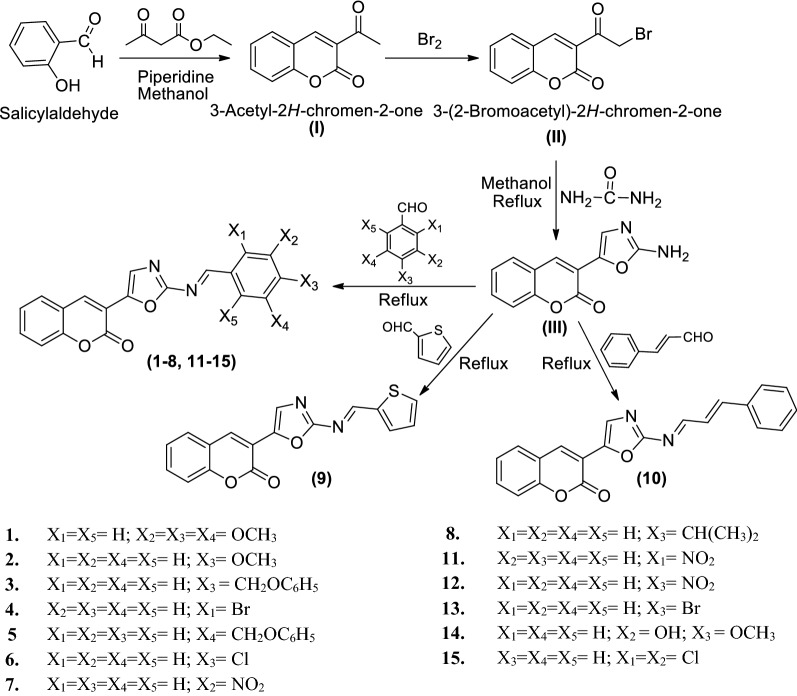
Synthesis of 3-(2-aminooxazol-5-yl)-2*H*-chromen-2-one derivatives (**1**–**15**)

The synthesis of oxazole derivatives (**1**–**15**) were accomplished using the synthetic procedure depicted in Scheme 1. At first, 3-acetyl-2*H*-chromen-2-one (**I**) was prepared by the reaction of salicylaldehyde and ethyl acetoacetate in the presence of piperidine. Further, the reaction of **I** with bromine resulted in the formation of 3-(2-bromoacetyl)-2*H*-chromen-2-one (**II**). The later was refluxed with urea to synthesize 3-(2-aminooxazol-5-yl)-2*H*-chromen-2-one (**III**). The reaction of 3-(2-aminooxazol-5-yl)-2*H*-chromen-2-one (**III**) with substituted aldehydes yielded the title compounds 3-(2-(substituted benzylideneamino)oxazol-5-yl)-2*H*-chromen-2-one derivatives (**1**–**15**). The physicochemical and spectral characteristics of the synthesized oxazole derivatives are given in Table 1. Spectral data (FT-IR (KBr, cm^−1^), ^1^H/^13^C–NMR (DMSO-*d*_6_, 600 MHz, δ ppm) and Mass spectral) studies helped in determining the molecular structures of the synthesized derivatives (**1**–**15**). The IR spectrum indicated that the appearance of bands at 3398–2924 cm^−1^, 1456–1415 cm^−1^, 1680–1595 cm^−1^, 1382–1236 cm^−1^ and 1724–1693 cm^−1^ displayed the presence of C–H, C=C, C=N, C–N and C=O groups, respectively in the synthesized compound. The absorption bands around 1292–1130 cm^−1^ corresponded to C–O–C stretching of oxazole compounds. In case of ^1^H-NMR spectra the presence of multiplet signals between 6.88 and 8.69 δ ppm reflected the presence of aromatic protons in synthesized derivatives. The compound **14** showed singlet (s) at 6.76 δ ppm because of the presence of OH of Ar–OH. The appearance of singlet (s) at 7.51–8.4 δ ppm and 6.9–7.37 δ ppm is due to the existence of N=CH and C–H of oxazole, respectively. Compound **8** showed multiplet and doublet signals at 3.11 δ ppm and 1.29 δ ppm due to existence of –CH and (CH_3_)_2_ groups of –CH(CH_3_)_2_ at the *para*-position. The compounds, **1**, **2** and **14** showed singlet at 3.73–3.89 δ ppm due to the existence of OCH_3_ of Ar–OCH_3_. The compounds, **3** and **5** showed singlet at 5.08 δ ppm due to the existence of –CH_2_–O group of (benzyloxy)benzene. The compound **10** displayed doublet signal at 5.59–6.95 δ ppm due to the existence of –CH=CH group of -prop-1-en-1-ylbenzene. The ^13^C–NMR spectrum indicated that the carbon signals around at 161.1, 128.5 (coumarin), 151.9 (N=CH), 136.1 (oxazole) of the synthesized compounds. Mass of synthesized compounds showed in (M^+^+1).

**Table 1:** The physicochemical and spectral characteristics of synthesized oxazole derivatives

Compound	Spectral characteristics
(**1**)	*(3*-*(2*-*(3,4,5*-*Trimethoxy*-*benzylidene*-*amino)oxazol*-*5*-*yl)*-*2H*-*chromen*-*2*-*one*): m.p. °C: 204–206; R*f* value: 0.35; % yield: 70; IR (KBr cm^−1^): 3100 (C–H str.), 1419 (C=C str.), 1606 (N=CH str.), 1236 (C–N str.), 1286 (C–O–C str.), 1722 (C=O str.), 2800 (OCH_3_ str.); ^1^H NMR (δ, DMSO): 7.22–7.54 (m, 7H, ArH), 8.39 (s, 1H, N=CH), 7.19 (s, 1H, CH of oxazole), 3.89 (s, 9H, (–OCH_3_)_3_); ^13^C NMR (δ, DMSO): 139.2 (oxazole-C), 128.1, 121.3, 120.2, 102.08 (phenyl nucleus), 55.8 (OCH_3_); M. Formula: C_22_H_18_N_2_O_6_; MS: *m/z* 407 (M^+^+1)
(**2**)	*3*-*(2*-*(4*-*Methoxybenzylidene*-*amino)oxazol*-*5*-*yl)*-*2H*-*chromen*-*2*-*one*): m.p. °C: 190–192; R*f* value: 0.34; % yield: 65; IR (KBr cm^−1^): 3174 (C–H str.), 1452 (C=C str.), 1595 (N=CH str.), 1292 (C–N str.), 1259 (C–O–C str.), 1724 (C=O str.), 3053 (OCH_3_ str.); ^1^H NMR (δ, DMSO): 6.94–7.92 (m, 9H, ArH), 8.17 (s, 1H, N=CH), 7.19 (s, 1H, CH of oxazole), 3.84 (s, 3H, –OCH_3_); ^13^C NMR (δ, DMSO): 163.8, 131.2, 114.7 (phenyl nucleus), 162.7, 128.8, 128.5, 127.2, 124.8 (coumarin-C), 158.3 (N=CH), 151.9, 137.7, 137.1 (oxazole-C), 55.6 (OCH_3_); M. Formula: C_20_H_14_N_2_O_4_; MS: *m/z* 347 (M^+^+1)
(**3**)	*(3*-*(2*-*(4*-*(Phenoxymethyl)*-*benzylideneamino)oxazol*-*5*-*yl)*-*2H*-*chromen*-*2*-*one*): m.p. °C: 186–188; R*f* value: 0.32; % yield: 72; IR (KBr cm^−1^): 3172 (C–H str.), 1450 (C=C str.), 1602 (N=CH str.), 1382 (C–N str.), 1257 (C–O–C str.), 1720 (C=O str.); ^1^H NMR (δ, DMSO): 7.00–7.93 (m, 14H, ArH), 8.3 (s, 1H, N=CH), 7.02 (s, 1H, CH of oxazole), 5.08 (s, 2H, –CH_2_–O); ^13^C NMR (δ, DMSO): 162.7, 127.8, 124.8 (coumarin-C), 161.1 (N=CH), 152.3, 137.7 (oxazole-C), 132.1, 128.8, 128.4, 127.4, 115.5 (phenyl nucleus), 69.6 (CH_2_O); M. Formula: C_26_H_18_N_2_O_4_; MS: *m/z* 423 (M^+^+1)
(**4**)	*(3*-*(2*-*(2*-*Bromobenzylidene*-*amino)oxazol*-*5*-*yl)*-*2H*-*chromen*-*2*-*one*): m.p. °C: 215–217; R*f* value: 0.48; % yield: 68; IR (KBr cm^−1^): 2937 (C–H str.), 1454 (C=C str.), 1602 (N=CH str.), 1292 (C–N str.), 1224 (C–O–C str.), 1722 (C=O str.), 592 (C–Br str.); ^1^H NMR (δ, DMSO): 7.25–7.83 (m, 9H, ArH), 7.84 (s, 1H, N=CH), 7.26 (s, 1H, CH of oxazole); ^13^C NMR (δ, DMSO): 135.1, 132.2, 131.3, 131.2, 120.4 (phenyl nucleus), 129.3, 128.6 (coumarin-C); M. Formula: C_19_H_11_BrN_2_O_3_; MS: *m/z* 396 (M^+^+1)
(**5**)	*(3*-*(2*-*(3*-*(Phenoxymethyl)*-*benzylideneamino)oxazol*-*5*-*yl)*-*2H*-*chromen*-*2*-*one*): m.p. °C: 184–186; R*f* value: 0.33; % yield: 75; IR (KBr cm^−1^): 3190 (C–H str.), 1450 (C=C str.), 1600 (N=CH str.), 1328 (C–N str.), 1292 (C–O–C str.), 1722 (C=O str.); ^1^H NMR (δ, DMSO): 7.16–7.69 (m, 14H, ArH), 8.4 (s, 1H, N=CH), 7.14 (s, 1H, CH of oxazole), 5.08 (s, 2H, –CH_2_–O); ^13^C NMR (δ, DMSO): 158.4, 140.2, 133.2, 128.4, 120.2, 115.6 (phenyl nucleus), 151.1, 140.5, 136.7 (oxazole-C), 129.7, 128.9, 128.4, 126.8, 125.5 (coumarin-C); M. Formula: C_26_H_18_N_2_O_4_; MS: *m/z* 423 (M^+^+1)
(**6**)	*(3*-*(2*-*(4*-*Chlorobenzylidenea*-*mino)oxazol*-*5*-*yl)*-*2H*-*chromen*-*2*-*one*): m.p. °C: 194–196; R*f* value: 0.29; % yield: 60; IR (KBr cm^−1^): 3070 (C–H str.), 1452 (C=C str.), 1600 (N=CH str.), 1328 (C–N str.), 1292 (C–O–C str.), 1724 (C=O str.); ^1^H NMR (δ, DMSO): 6.89–7.68 (m, 9H, ArH), 8.11 (s, 1H, N=CH), 7.37 (s, 1H, CH of oxazole); ^13^C NMR (δ, DMSO): 161.1, 129.3, 128.5, 124.8, 119.1 (coumarin-C), 158.3 (N=CH), 151.9 (oxazole-C), 136.1, 131.2 (phenyl nucleus); M. Formula: C_19_H_11_ClN_2_O_3_; MS: *m/z* 351 (M^+^+1)
(**7**)	*(2*-*(3*-*Nitrobenzylideneamino)*-*oxazol*-*5*-*yl)*-*2H*-*chromen*-*2*-*one*): m.p. °C: 236–238; R*f* value: 0.51; % yield: 79; IR (KBr cm^−1^): 2972 (C–H str.), 1454 (C=C str.), 1606 (N=CH str.), 1276 (C–N str.), 1130 (C–O–C str.), 1714 (C=O str.), 1344 (NO_2_ str.); ^1^H NMR (δ, DMSO): 6.90–8.69 (m, 9H, ArH), 7.98 (s, 1H, N=CH), 7.14 (s, 1H, CH of oxazole); ^13^C NMR (δ, DMSO); 148.2, 134.8, 130.9 (phenyl nucleus), 137.1 (oxazole-C), 129.7, 128.4, 123.9 (coumarin-C); M. Formula: C_19_H_11_N_3_O_5_; MS: *m/z* 362 (M^+^+1)
(**8**)	*(3*-*(2*-*(4*-*Isopropylbenzylidene*-*amino)oxazol*-*5*-*yl)*-*2H*-*chromen*-*2*-*one*): m.p. °C: 206–208; R*f* value: 0.39; % yield: 80; IR (KBr cm^−1^): 3398 (C–H str.), 1415 (C=C str.), 1604 (N=CH str.), 1253 (C–N str.), 1157 (C–O–C str.), 1720 (C=O str.); ^1^H NMR (δ, DMSO): 6.88–7.84 (m, 9H, ArH), 8.12 (s, 1H, N=CH), 7.37 (s, 1H, CH of oxazole), {3.11 (m, 1H, CH of –CH(CH_3_)_2_), 1.29 (d, 6H, (CH_3_)_2_)}; ^13^C NMR (δ, DMSO): 161.1, 128.5, 119.1 (coumarin-C), 158.3 (N=CH), 151.9, 131.2, 124.6 (phenyl nucleus), 136.1 (oxazole-C); M. Formula: C_22_H_18_N_2_O_3_; MS: *m/z* 359 (M^+^+1)
(**9**)	*(3*-*(2*-*(Thiophen*-*2*-*ylmethylene*-*amino)oxazol*-*5*-*yl)*-*2H*-*chromen*-*2*-*one*): m.p. °C: 179–181; R*f* value: 0.49; % yield: 75; IR (KBr cm^−1^): 3118 (C–H str.), 1454 (C=C str.), 1604 (N=CH str.), 1274 (C–N str.), 1253 (C–O–C str.), 1693 (C=O str.), 715 (C-S str.); ^1^H NMR (δ, DMSO): 7.38–7.84 (m, 5H, ArH), 7.59 (s, 1H, N=CH), 6.9 (s, 1H, CH of oxazole), {7.6 (d, 1H, CH), 7.17 (t, 1H, CH), 7.68 (d, 1H, CH) of thiophene}; ^13^C NMR (δ, DMSO): 161.1, 128.5 (coumarin-C), 151.9 (N=CH), 136.1 (oxazole-C), 124.6 (thiophene-C); M. Formula: C_17_H_10_N_2_O_3_S; MS: *m/z* 323 (M^+^+1)
(**10**)	*(3*-*(2*-*3*-*Phenylallylidene)*-*amino)*-*oxazol*-*5*-*yl)*-*2H*-*chromen*-*2*-*one*): m.p. °C: 210–212; R*f* value: 0.52; % yield: 65; IR (KBr cm^−1^): 2924 (C–H str.), 1456 (C=C str.), 1680 (N=CH str.), 1294 (C–N str.), 1226 (C–O–C str.), 1710 (C=O str.), 1606 (C=C con); ^1^H NMR (δ, DMSO): 7.10–7.75 (m, 10H, ArH), 7.51 (s, 1H, N=CH), 7.09 (s, 1H, CH of oxazole), 5.59–6.95 (d, 2H, –CH=CH); ^13^C NMR (δ, DMSO): 150.9, 141.1 (oxazole-C), 128.7, 128.6, 128.2 (phenyl nucleus), 128.5, 127.1, 123.6 (coumarin-C); M. Formula: C_21_H_14_N_2_O_5_; MS: *m/z* 343 (M^+^+1)
(**11**)	(*3*-*(2*-*(2*-*Nitrobenzylideneam*-*ino)oxazol*-*5*-*yl)*-*2H*-*chromen*-*2*-*one*): m.p. °C: 248–250; R*f* value: 0.42; % yield: 68; IR (KBr cm^−1^): 3369 (C–H str.), 1454 (C=C str.), 1604 (N=CH str.), 1274 (C–N str.), 1130 (C–O–C str.), 1703 (C=O str.), 1342 (NO_2_ str.); ^1^H NMR (δ, DMSO): 7.24–7.58 (m, 9H, ArH), 7.92 (s, 1H, N=CH), 7.23 (s, 1H, CH of oxazole); ^13^C NMR (δ, DMSO): 138.1 (oxazole-C), 137.1, 131.9, 130.3 (phenyl nucleus), 128.1, 126.1, 122.3, 121.5 (coumarin-C); M. Formula: C_19_H_11_N_3_O_5_; MS: *m/z* 362 (M^+^+1)
(**12**)	*(3*-*(2*-*(4*-*Nitrobenzylideneam*-*ino)oxazol*-*5*-*yl)*-*2H*-*chromen*-*2*-*one*): m.p. °C: 236–238; R*f* value: 0.37; % yield: 74; IR (KBr cm^−1^): 2972 (C–H str.), 1454 (C=C str.), 1604 (N=CH str.), 1274 (C–N str.), 1170 (C–O–C str.), 1714 (C=O str.), 1340 (NO_2_ str.); ^1^H NMR (δ, DMSO): 6.89–8.23 (m, 9H, ArH), 8.16 (s, 1H, N=CH), 7.17 (s, 1H, CH of oxazole); ^13^C NMR (δ, DMSO): 131.3, 124.3 (coumarin-C), 130.5, 115.9 (phenyl nucleus); M. Formula: C_19_H_11_N_3_O_5_; MS: *m/z* 362 (M^+^+1)
(**13**)	*(3*-*(2*-*(4*-*Bromobenzylidene*-*amino)oxazol*-*5*-*yl)*-*2H*-*chromen*-*2*-*one*): m.p. °C: 179–181; R*f* value: 0.39; % yield: 63; IR (KBr cm^−1^): 3070 (C–H str.), 1452 (C=C str.), 1606 (N=CH str.), 1274 (C–N str.), 1192 (C–O–C str.), 1722 (C=O str.), 592 (C–Br str.); ^1^H NMR (δ, DMSO): 7.05–7.81 (m, 9H, ArH), 7.85 (s, 1H, N=CH), 7.06 (s, 1H, CH of oxazole); ^13^C NMR (δ, DMSO): 135.1 (oxazole-C), 132.2, 131.3, 131.1 (phenyl nucleus), 129.3, 129.1, 124.6 (coumarin-C); M. Formula: C_19_H_11_BrN_2_O_3_; MS: *m/z* 396 (M^+^+1)
(**14**)	*(3*-*(2*-*(3*-*Hydroxy*-*4*-*methoxy*-*benzylideneamino)oxazol*-*5*-*yl)*-*2H*-*chromen*-*2*-*one*): m.p. °C: 228–230; R*f* value: 0.46; % yield: 78; IR (KBr cm^−1^): 3178 (C–H str.), 1454 (C=C str.), 1606 (N=CH str.), 1259 (C–N str.), 1192 (C–O–C str.), 1722 (C=O str.), 2935 (OCH_3_ str.); 3408 (OH); ^1^H NMR (δ, DMSO): 7.18–7.71 (m, 8H, ArH), 8.07 (s, 1H, N=CH), 7.20 (s, 1H, CH of oxazole), 6.76 (s, 1H, –OH), 3.73 (s, 3H, –OCH_3_); ^13^C NMR (δ, DMSO): 160.2 (N=CH), 154.3, 127.5, 116.2, 115.8 (phenyl nucleus), 151.1, 140.8, 139.4 (oxazole-C), 128.3, 124.8, 120.1 (coumarin-C); M. Formula: C_20_H_14_N_3_O_5_; MS: *m/z* 363 (M^+^+1)
(**15**)	*(3*-*(2*-*(2,3*-*Dichlorobenzyli*-*deneamino)oxazol*-*5*-*yl)*-*2H*-*chromen*-*2*-*one*): m.p. °C: 219–221; R*f* value: 0.44; % yield: 74; IR (KBr cm^−1^): 3072 (C–H str.), 1452 (C=C str.), 1604 (N=CH str.), 1253 (C–N str.), 1192 (C–O–C str.), 1722 (C=O str.), 750 (C–Cl str.); ^1^H NMR (δ, DMSO): 7.30–7.88 (m, 8H, ArH), 8.14 (s, 1H, N=CH), 7.30 (s, 1H, CH of oxazole); ^13^C NMR (δ, DMSO): 131.5, 131.2 (phenyl nucleus), 128.8 (coumarin-C); M. Formula: C_19_H_10_Cl_2_N_2_O_3_; MS: *m/z* 386 (M^+^+1)

### Antimicrobial activity

**Fig. 3:**
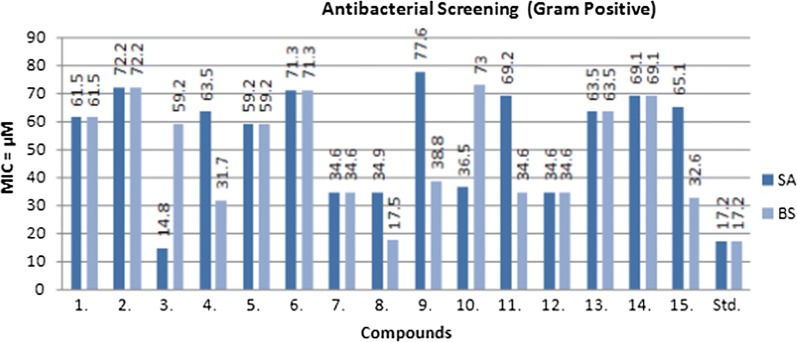
Antibacterial screening results against Gram positive species

**Fig. 4:**
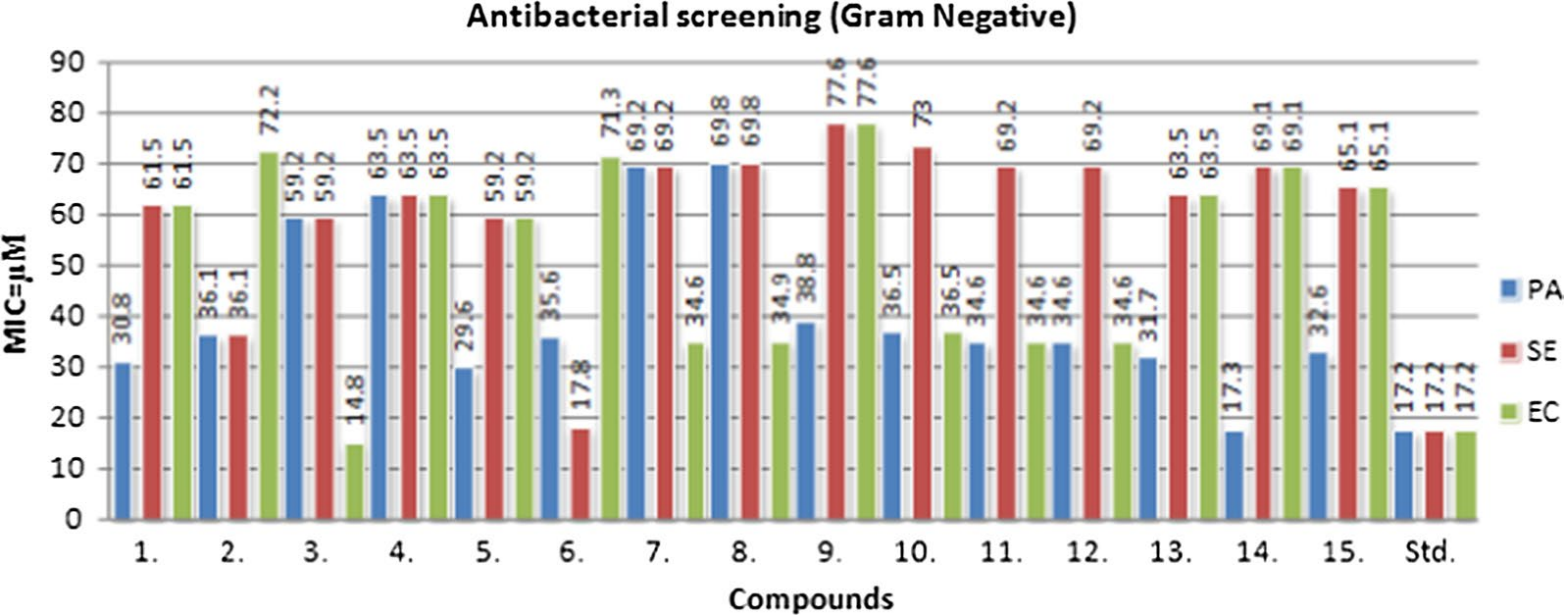
Antibacterial screening results against Gram negative species

**Fig. 5:**
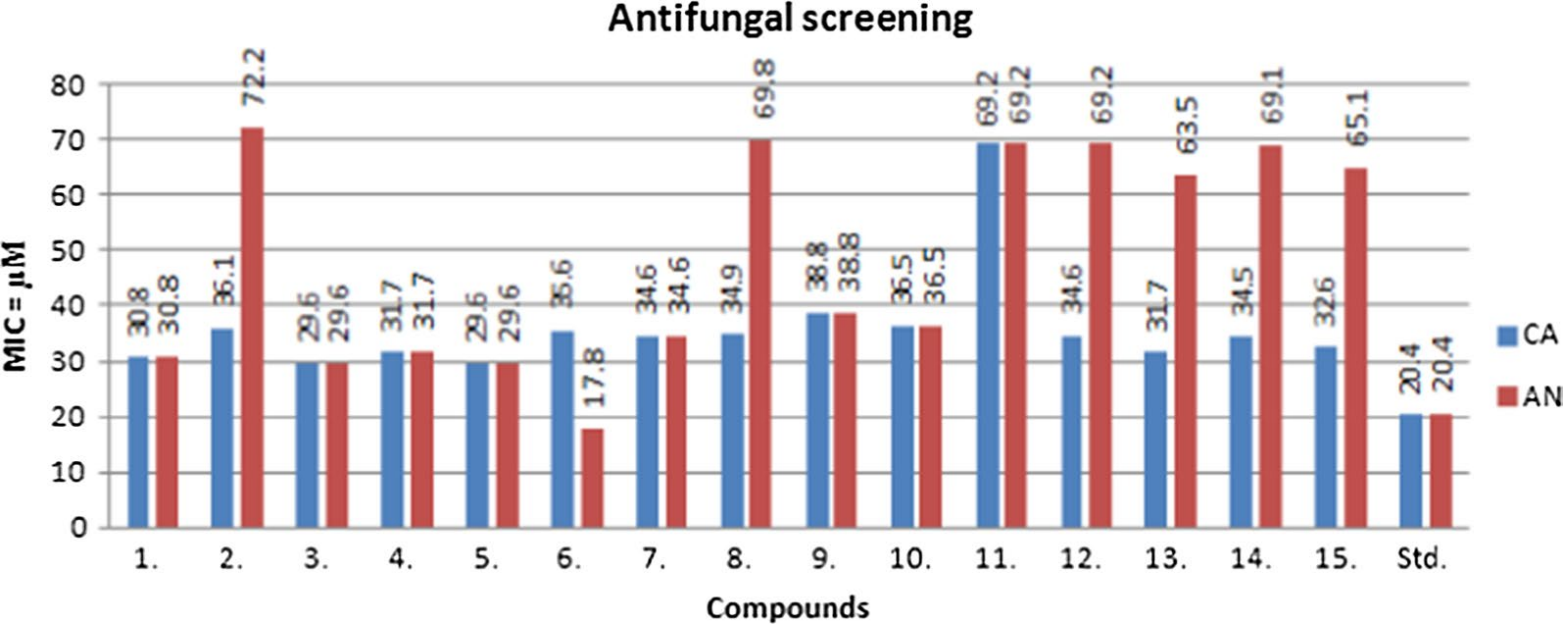
Antifungal screening results against fungal species

The in vitro antimicrobial potential of the prepared oxazole derivatives was determined by tube dilution technique (Table 2, Fig. 3, 4 and 5). The antibacterial screening results revealed that compound **3** was moderately potent against *S. aureus* with MIC_*sa*_ value of 14.8 µM and compound **8** was moderately active against *B. subtilis* with MIC_*bs*_ value of 17.5 µM. Compound **3** (MIC_*ec*_ = 14.8 µM) was found to be effective against *E. coli*. Compound **14** (MIC_*pa*_ = 17.3 µM) and compound **6** (MIC_*se*_= 17.8 µM) exhibited promising activity against *P. aeruginosa* and *S. enterica,* respectively. The antifungal activity results indicated that compound **6** (MIC_*an*_ = 17.8 µM) displayed most potent activity against *A. niger* and compounds **3** and **5** (MIC_*ca*_= 29.6 µM) were found to be moderately potent against *C. albicans.* The antibacterial screening results are comparable to the standard drug (cefadroxil), whereas antifungal results of compound **6** showed less activity against *A. niger* and compound **5** showed more against *C. albicans* than the standard drug (fluconazole) and these compounds may be used as a lead compound to discover novel antimicrobial agents.

**Table 2:** In vitro antimicrobial activity of the synthesized compounds

Comp.	Antimicrobial screening (MIC = µM)						
	SA	EC	BS	PA	SE	CA	AN
**1**	61.5	61.5	61.5	30.8	61.5	30.8	30.8
**2**	72.2	72.2	72.2	36.1	36.1	36.1	72.2
**3**	14.8	14.8	59.2	59.2	59.2	29.6	29.6
**4**	63.5	63.5	31.7	63.5	63.5	31.7	31.7
**5**	59.2	59.2	59.2	29.6	59.2	29.6	29.6
**6**	71.3	71.3	71.3	35.6	17.8	35.6	17.8
**7**	34.6	34.6	34.6	69.2	69.2	34.6	34.6
**8**	34.9	34.9	17.5	69.8	69.8	34.9	69.8
**9**	77.6	77.6	38.8	38.8	77.6	38.8	38.8
**10**	36.5	36.5	73.0	36.5	73.0	36.5	36.5
**11**	69.2	34.6	34.6	34.6	69.2	69.2	69.2
**12**	34.6	34.6	34.6	34.6	69.2	34.6	69.2
**13**	63.5	63.5	63.5	31.7	63.5	31.7	63.5
**14**	69.1	69.1	69.1	17.3	69.1	34.5	69.1
**15**	65.1	65.1	32.6	32.6	65.1	32.6	65.1
Cefadroxil	17.2	17.2	17.2	17.2	17.2	–	–
Fluconazole	–	–	–	–	–	20.4	20.4

*SA, Staphylococcus aureus, EC, Escherichia coli; BS, Bacillus subtilis; PA, Pseudomonas aeruginosa; SE, Salmonella enterica; CA, Candida albicans; AN, Aspergillus niger*

### Anticancer activity

The synthesized derivatives were also screened for their cytotoxic effect using Sulforhodamine B (SRB) assay [26] against two cancer cell lines- human colorectal carcinoma (HCT116) and oestrogen-positive human breast carcinoma (MCF7). In the case of HCT116, compound **14** exhibited good activity with IC_50_ = 71.8 µM. In the case of MCF7, compound **6** exhibited good activity with IC_50_ = 74.1 µM. Reference drugs used in the study were 5-flourouracil (for HCT116) and tamoxifen (MCF7). They had yielded IC_50_ values of 12.7 µM and 4.3 µM, respectively and these compounds may be used as a lead compound to discover novel anticancer agents. Results are displayed in Table 3.

**Table 3:** In vitro anticancer screening of the synthesized compounds

Comp.	Anticancer screening (IC_50_ = µM)	
	Cancer cell lines	
	HCT116	MCF7
**1**	221.5	> 246.1
**2**	288.7	> 288.7
**3**	> 236.7	> 236.7
**4**	> 253.8	> 253.8
**5**	> 236.9	> 236.9
**6**	> 285.1	74.1
**7**	> 277.0	207.7
**8**	203.8	> 279.2
**9**	> 310.2	263.7
**10**	192.8	262.9
**11**	> 276.8	> 276.8
**12**	221.4	83.0
**13**	> 253.8	> 253.8
**14**	71.8	193.4
**15**	> 260.4	> 260.4
5-Fluorouracil	12.7	–
Tamoxifen	–	4.3

### Molecular docking results

The mammalian cyclin-dependent kinase 8 (cdk8) protein which is a component of the RNA polymerase has been one of the proteins responsible for acute lymphoblastic leukaemias. CDK-8 is a heterodimeric kinase protein responsible for regulation of cell cycle progression, transcription and other functions. CDK-8 phosphorylates the carboxyterminal domain of the largest subunit of RNA polymerase II like protein kinases. Therefore, the inhibition of CDK-8 protein may be crucial for controlling cancer [27]. Since compounds were screened through ATP binding pocket so, ATP was used as docking control to compare the binding affinity of compounds within the binding pocket. The synthesized oxazole compounds showed good docking score and were found to interact with important amino acids for the biological function of CDK-8 protein.

**Fig. 6:**
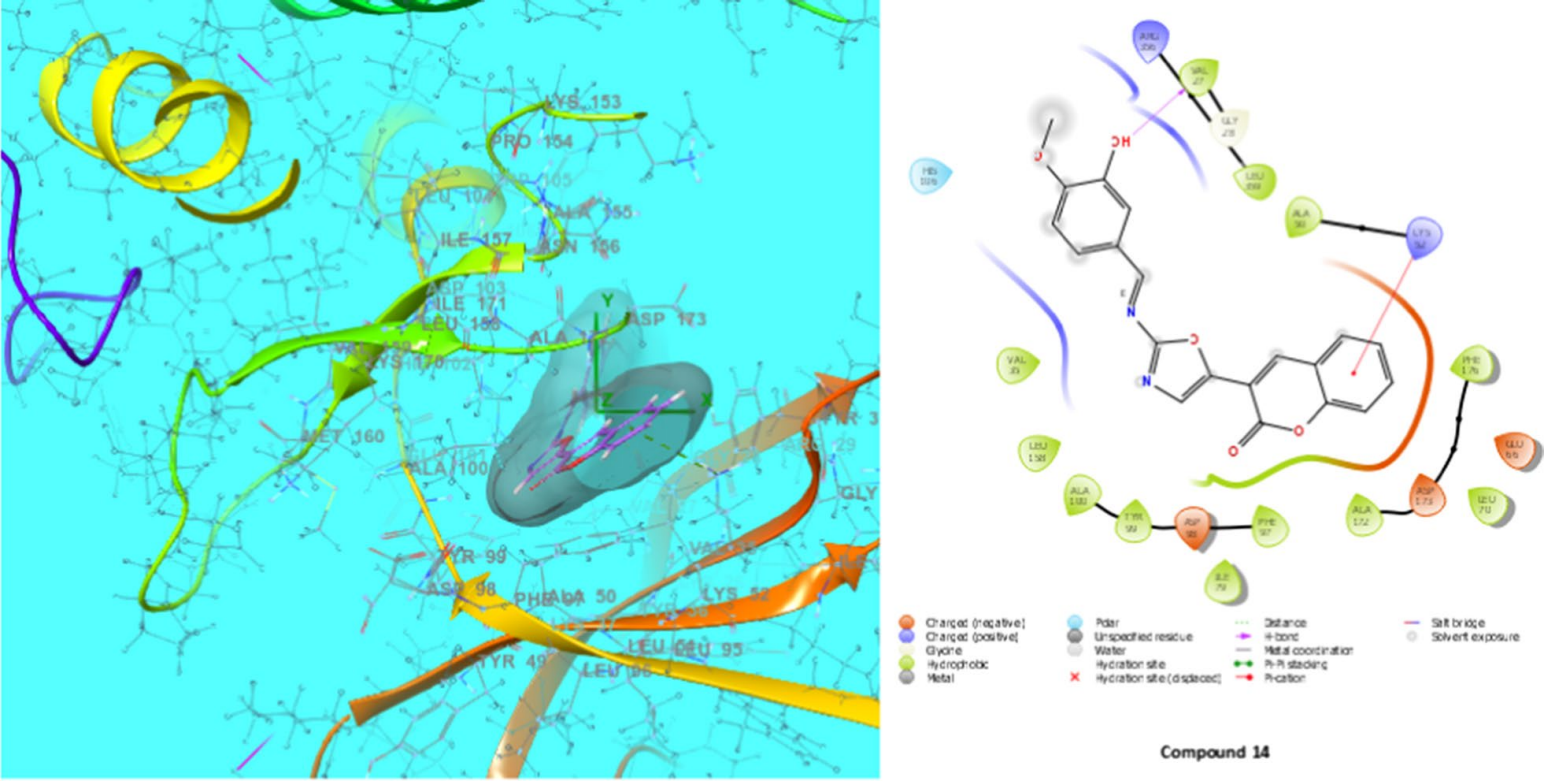
Interaction of compound **14** and 5-fluorouracil within the active pocket of cdk-8 protein and interacting amino acid in 2D view

**Fig. 7:**
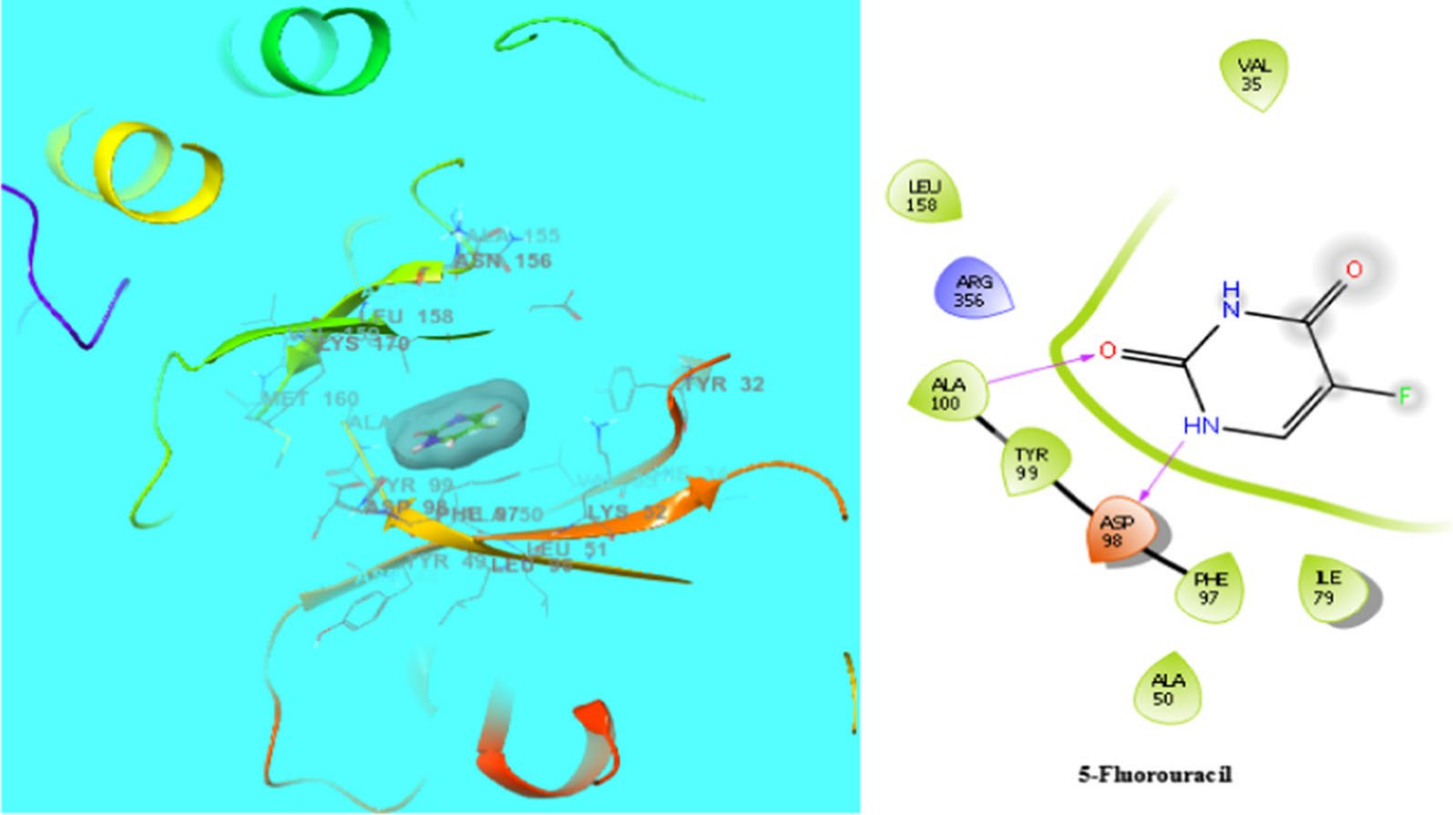
Interaction of 5-fluorouracil within the active pocket of cdk-8 protein and interacting amino acid in 2D view

Molecular docking were carried out to analyse the binding mode of the most active compound **14** and compound **6** against human colorectal carcinoma HCT116 and oestrogen- positive human breast carcinoma MCF7 cancer cell lines respectively. The molecular docking study was carried out on GLIDE docking program. The compound **14** was docked in the active site of the cyclin dependent kinase cdk8 (PDB: 5FGK) co-crystallized wit 5XG ligand. The results were analysed based on the docking score obtained from GLIDE. Ligand interaction diagram and displayed the binding mode of compound **14** in the active site of cdk8 having co cystallised ligand 5XG and 5-fluorouracil (the standard inhibitor of cancer) is having a different binding mode to that of active compound (Figs. 6 and 7).

**Fig. 8:**
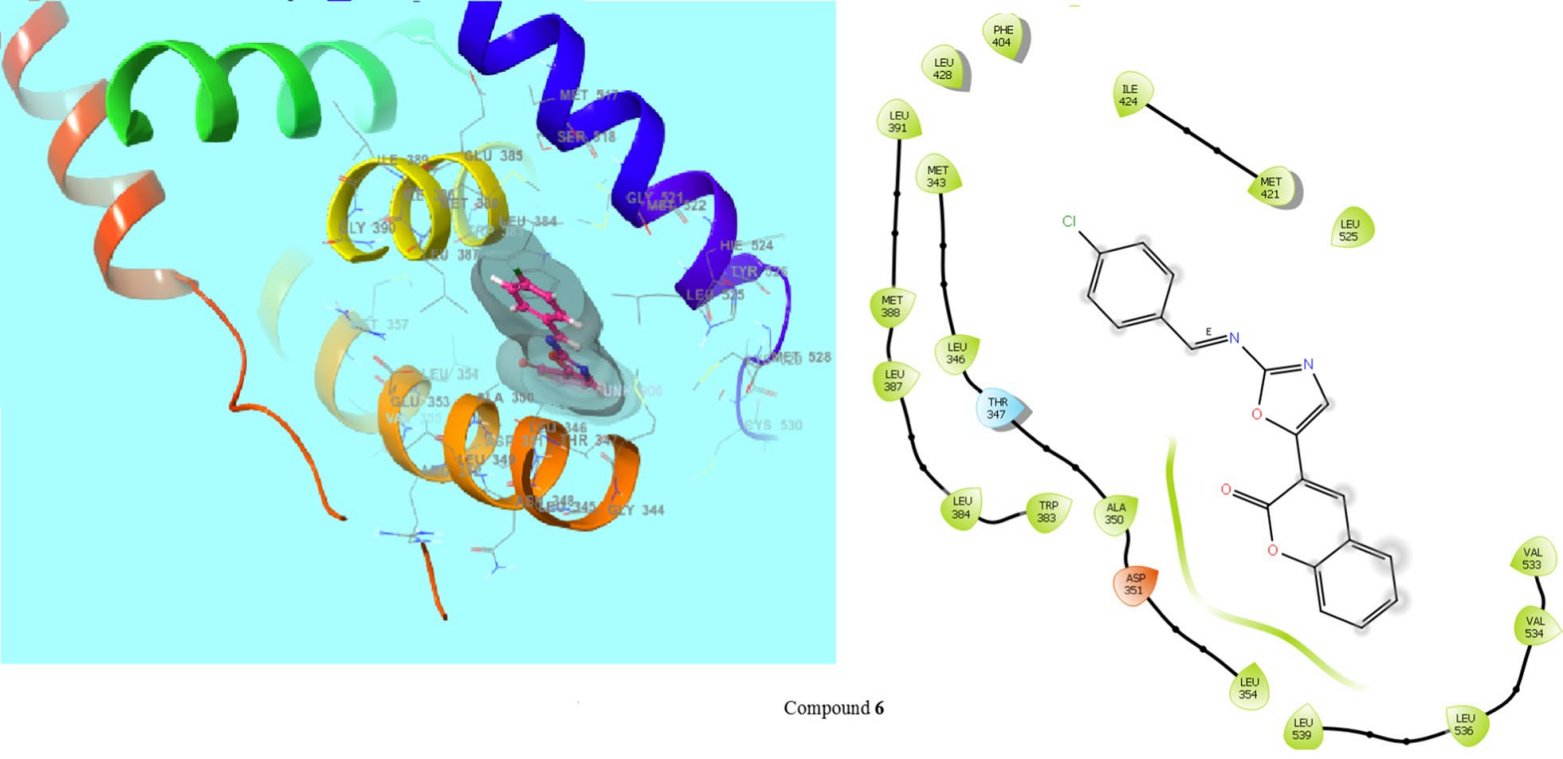
Interaction of compound **6** and tamoxifen within the active pocket of 3ERT protein and interacting amino acid in 2D view

**Fig. 9:**
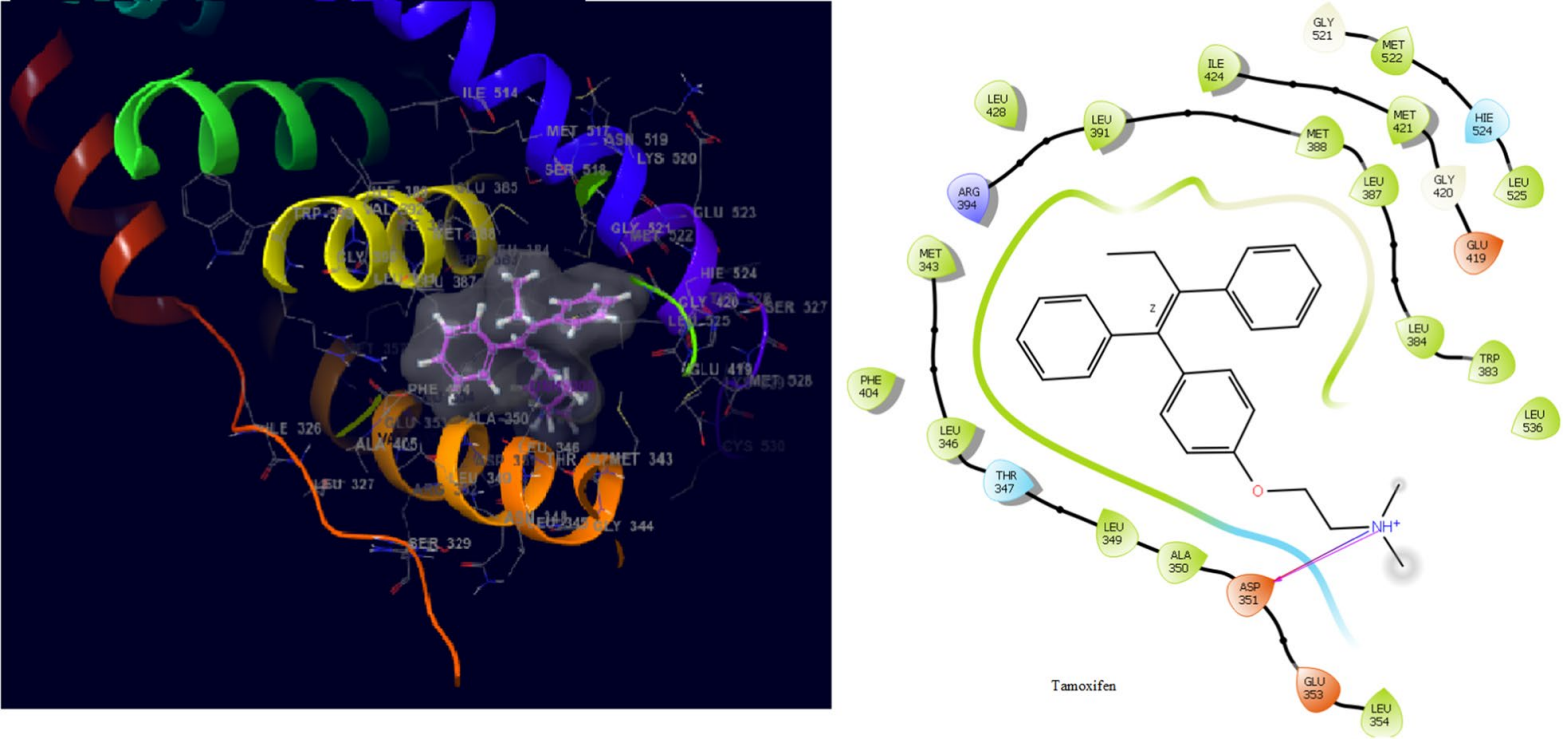
Interaction of tamoxifen within the active pocket of 3ERT protein and interacting amino acid in 2D view

The compound **6** was docked in the active site of the ER-alpha of MCF-7 (PDB: 3ERT) co-crystallized wit OHT (Tamoxifen) ligand. The results were analysed based on the docking score obtained from GLIDE. Ligand interaction diagram and show the binding mode of compound **6** in the active site of ER apha having co cystallised ligand OHT and Tamoxifen (the standard inhibitor of cancer) is having a different binding mode to that of active compound (Figs. 8 and 9). The docking scores were demonstrated in terms of negative energy; the lower the binding energy, best would be the binding affinity. The results depend on the statistical evaluation function according to which the interaction energy in numerical values as docking scores. The 3D pose of the ligand interaction with receptor can be visualized using different visualization tools [28]. Based on the molecular docking study the selected compounds with good anticancer activity against cancer cell lines (HCT116 and MCF-7) were displayed good interaction with crucial amino acids. Like if we look into the best-fitted compound **14** showed the best dock score (− 7.491) with better potency (71.8 µM) within the ATP binding pocket (Table 4). Compound **6** showed the best dock score (− 6.462) with better potency (74.1 µM) within the ATP binding pocket (Table 5). Thus the docking results suggest that the oxazole derivatives can act as of great interest in successful chemotherapy. CDK-8 may be the target protein of oxazole derivatives for their anticancer activity at lower micromolar concentrations. Based on the docking analysis it is suggested that more structural modifications are required in compounds **6** and **14** to make them more active against cancer cells and to have activity comparable to the standards 5-fluorouracil and tamoxifen.

**Table 4:** Docking score and binding energy of compound **14** with standard drug (5-fluorouracil)

Compound	Docking score	Interacting residues
14	− 7.491	ARG356, VAL27, GLY28, LEU359, ALA50, LYS52, VAL35, LEU158, ASP98, PHE97, ALA172, ASP173, PHE176, ALA100, TYR99
5-fluorouracil	− 5.753	LEU158, ARG356, ALA100, TYR99, ASP98, PHE97, ILE79, VAL35, ALA50

**Table 5:** Docking score and binding energy of compound **6** with standard drug (tamoxifen)

Compound	Docking score	Interacting residues
6	− 6.462	ILE424, MET421, LEU525, MET343, LEU346, THR347, A350, ASP351, LEU354, LEU539, LEU536, VAL534, VAL533
Tamoxifen	− 11.595	ASP351, GLU353, LEU354, ALA350, LEU349, THR347, LEU346, MET343, ARG394, LEU391, MET388, LEU387, LEU384, TRP383, LEU536

#### Structure activity relationship

**Fig. 10:**
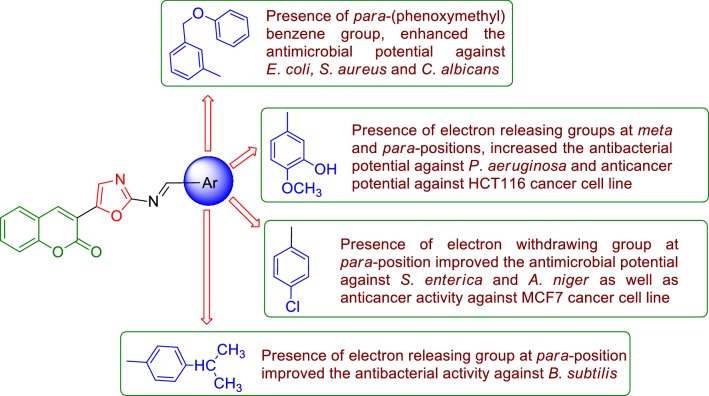
Structure activity relationship of synthesized compounds

From the antimicrobial and anticancer activities results following structure activity can be derived (Fig. 10):

- The different substitution of aldehydes were used to synthesized the final derivatives of 3-(2-aminooxazol-5-yl)-2*H*-chromen-2-one derivatives played an important role in improving the antimicrobial and anticancer activities. Presence of electron releasing group (–CH(CH_3_)_2_) at *para* position of the substitution part of the synthesized compound **8**, increased the antibacterial activity against *B. subtilis.* Presence of *para*-(phenoxy-methyl)benzene group (compound **3**), enhanced the antibacterial activity against *E. coli* and *S. aureus* as well antifungal activity against *C. albicans* whereas (Compound **5**) also improved the antifungal activity against *C. albicans.*
- Presence of electron releasing group (OH, OCH_3_) at *meta* and *para* position of the substitution portion of the synthesized compound **14**, increased the antibacterial activity against *P. aeruginosa* and also increased anticancer activity against HCT116 cancer cell line whereas electron withdrawing groups (–Cl) at *para*-position of the synthesized compound **6,** improved the antimicrobial activity against *S. enterica* and *A. niger* as well as anticancer activity against MCF7 cancer cell line. These compounds may be used as a lead compound to discover novel antimicrobial and anticancer agents.

### Experimental part

The chemicals procured were of analytical grade and were further used without any purification. Melting point (m.p.) was determined in open glass capillaries on a Stuart scientific SMP3 apparatus. Reaction progress of every synthetic step was confirmed by TLC plates on silica gel sheets. ^1^H and ^13^C–NMR spectra were determined by Bruker Avance III 600 NMR spectrometer in appropriate deuterated solvents and are expressed in parts per million (δ, ppm) downfield from tetramethylsilane (internal standard). Proton NMR spectra are given as multiplicity (s, singlet; d, doublet; t, triplet; m, multiplet) and number of protons. Infrared (IR, KBr, cm^−1^) spectra were recorded as KBr pellets on Shimadzu FTIR 8400 spectrometer. Waters Micromass Q-ToF Micro instrument was used for obtaining the Mass spectra.

### Synthetic steps of Scheme 1

*Step 1: Synthesis of 3-acetyl-2H-chromen-2-one (****I****)* To a solution of salicylaldehyde (0.025 mol) and ethyl acetoacetate (0.025 mol) in methanol (15 mL), 2–3 drops of piperidine was added, shaken with stirring and allowed to stand at room temperature for 30 min. Needle shaped crystals of 3-acetyl-2*H*-chromen-2-one (**I**) were obtained which were filtered dried and recrystallized from methanol [29].

*Step 2: Synthesis of 3-(2-bromoacetyl)-2H-chromen-2-one (****II****)* To a solution of 3-acetyl-2*H*-chromen-2-one (0.01 mol) in chloroform (15 mL), bromine (1.7 g) in chloroform (6 mL), was added with intermittent shaking and warming. The mixture was heated on water bath for 15 min to expel most of hydrogen bromide. The solution was cooled, filtered and recrystallized from acetic acid so as to obtain 3-(2-bromoacetyl)-2*H*-chromen-2-one (**II**) [29].

*Step 3: Synthesis of 3-(2-aminooxazol-5-yl)-2H-chromen-2-one (****III****)* To the methanolic solution of compound **II** (0.01 mol), urea (0.01 mol) was added. The reaction mixture was refluxed for 12 h, poured on to crushed ice and resultant solid was recrystallized with methanol to obtain **III** [30].

*Step 4: Synthesis of title compounds (****1****–****15****)* To the solution of compound **III** (0.01 mol) in methanol (50 mL), different substituted aldehydes (0.01 mol) were added and refluxed for 12 h. The reaction mixture was concentrated to half of its volume after refluxing and poured onto crushed ice. The resulting solution was then evaporated and the residue thus obtained was washed with water and finally recrystallized from methanol to give final compounds (**1**–**15**).

### In vitro antimicrobial assay

Tube dilution method [31] was used for evaluating the antimicrobial potential of the compounds and the standard drugs used were cefadroxil (antibacterial) and fluconazole (antifungal). The microbial species used in the study were Gram +ve and Gram −ve bacteria, i.e. MTCC-441 (*B. subtilis*), MTCC-3160 (*S. aureus*), MTCC-424 (*P. aeruginosa*), MTCC 1165 (*S. enterica*) and MTCC-443 (*E. coli*). The antifungal potential was assessed against MTCC-227 (*C. albicans*), and MTCC-281 (*A. niger*). Double strength nutrient broth I.P. (bacteria) or sabouraud dextrose broth I.P. (fungi) [32] were used for antimicrobial study. Dimethyl sulfoxide was used for preparing the stock solution of the test and reference compounds. Results were noted in MIC after incubating the samples at 37 ± 1 °C (24 h) for bacteria, at 25 ± 1 °C (7 days) for *A. niger* and at 37 ± 1 °C (48 h) for *C. albicans*, respectively. The lowest concentration of the tested compound that showed no visible growth of microorganisms in the test tube was noted as MIC.

### In vitro anticancer assay

The cytotoxic effect of oxazole derivatives was determined against two different cancer cell lines—human colorectal carcinoma [HCT116] and oestrogen- positive human breast carcinoma (MCF7) using Sulforhodamine-B assay. HCT116 was seeded at 2500 cells/well (96 well plate) whereas MCF7 was seeded at 3000 cells/well (96 well plate). The cells were allowed to attach overnight before being exposed to the respective compounds for 72 h. The highest concentration of each compound tested (100 µg/mL) contained only 0.1% DMSO (non-cytotoxic). Sulforhodamine B (SRB) assay [26] was then performed. Trichloroacetic acid was used for fixing the cells and then staining was performed for 30 min with 0.4% (w/v) sulforhodamine B mixed with 1% acetic acid. After five washes of 1% acetic acid solution, protein-bound dye was extracted with 10 mM tris base solution. Optical density was read at 570 nm and IC_50_ (i.e. concentration required to inhibit 50% of the cells) of each compound was determined. Data was presented as mean IC_50_ of at least triplicates.

### Molecular docking study

The protein target for oxazole derivatives was identified through the literature. Since the oxazole nucleus has vast medicinal properties, so the targets enzymes/receptors were found targeted with anticancer effect of oxazole compounds were collected for selection [33]. Cyclin-dependent kinase-8 (PDB Id: 5FGK) co-crystallized wit 5XG ligand and ER-alpha of MCF-7 (PDB: 3ERT) co-crystallized wit OHT (Tamoxifen) ligand excellent target against cancer [34], was retrieved from Protein Data Bank (http://www.rcsb.org/pdb/home/home.do) for docking of potent synthesized oxazole compounds. Docking score obtained from GLIDE and ATP binding site was targeted and the grid was created. The active site grid covered the important amino acids interacting with ATP [35].

## Conclusion

A series of oxazole derivatives was designed, synthesized and evaluated for its antimicrobial and antiproliferative activities. The biological screening results indicated that the compounds **3**, **5**, **6**, **8** and **14** had the best antimicrobial activity and had MIC values comparable to the standard drugs whereas in the case of anticancer activity, compound **14** was found to be moderate activity against HCT116 while compounds **6** was moderate activity against MCF7. Further molecular docking study indicated that compound **14** showed the best dock score (− 7.491) with better potency (71.8 µM) within the ATP binding pocket. Compound **6** showed the best dock score (− 6.462) with better potency (74.1 µM) within the ATP binding pocket. Hence these compounds may be taken as lead compound for further development of novel antimicrobial and anticancer agents.
